# Assessing the Antibacterial Potential and Biofilm Inhibition Capability of Atorvastatin-Loaded Nanostructured Lipid Carriers via Crystal Violet Assay

**DOI:** 10.3390/ph18030417

**Published:** 2025-03-15

**Authors:** Njoud Altuwaijri, Rawan Fitaihi, Fai A. Alkathiri, Sarah I. Bukhari, Alanoud M. Altalal, Alyaa Alsalhi, Lama Alsulaiman, Aljawhara O. Alomran, Noura S. Aldosari, Safa A. Alqhafi, Majd Alhamdan, Rihaf Alfaraj

**Affiliations:** 1 Department of Pharmaceutics, College of Pharmacy, King Saud University, Riyadh 11451, Saudi Arabia; naltuwaijri@ksu.edu.sa (N.A.); falkathire@ksu.edu.sa (F.A.A.); sbukhari@ksu.edu.sa (S.I.B.); alanoud@ksu.edu.sa (A.M.A.); asalsalhi@ksu.edu.sa (A.A.); laalsulaiman9@gmail.com (L.A.); omranj1@gmail.com (A.O.A.); salqhafi@ksu.edu.sa (S.A.A.); majd.m.alhamdan@gmail.com (M.A.); ralfaraj@ksu.edu.sa (R.A.); 2 Department of Botany and Microbiology, College of Science, King Saud University, Riyadh 11451, Saudi Arabia; nsaldosari@ksu.edu.sa

**Keywords:** atorvastatin, nanostructured lipid carriers (NLC), antibacterial activity, biofilm inhibition

## Abstract

**Background/Objectives**: Atorvastatin (ATR), an antihyperlipidemic drug with a potential antibacterial effect, was investigated in this study. Like other statins, ATR has been repurposed for several uses, ranging from anti-inflammatory to antimicrobial applications, and has demonstrated successful results. However, the efficacy of ATR is limited by its low solubility, indicating an opportunity for its encapsulation in a nanotechnology-based drug delivery system. **Methods**: Nanostructured lipid carrier (NLC) formulations were prepared using high-pressure homogenization and ultrasonication. The formulations were characterized, including their particle size, polydispersity index, zeta potential, encapsulation efficiency, and in vitro release. Antibacterial activity against methicillin-resistant *Staphylococcus aureus* (MRSA), *Escherichia coli* (*E. coli*), and *Staphylococcus aureus* (*S. aureus*) was evaluated using the growth curve (bacterial growth over time) and well diffusion methods (zone of inhibition and minimum inhibitory concentration (MIC) determination). The crystal violet assay was employed to assess biofilm inhibition. **Results**: The NLC formulations were optimized, and the size and zeta potential of the blank nanoparticles were 130 ± 8.39 nm and −35 ± 0.5 mV, respectively. In comparison, the encapsulated NLCs had a size of 142 ± 52.20 nm and a zeta potential of −31 ± 1.41 mV. The average encapsulation efficiency was 94%, and 70% of the drug was released after 24 h. The ATR-loaded NLCs showed significantly enhanced antibacterial activity by reducing the minimum inhibitory concentration by 2.5-fold for *E. coli*, 1.8-fold for *S. aureus*, and 1.4-fold for MRSA, and promoting more effective bacterial growth inhibition. Notably, biofilm inhibition was significantly improved with ATR-NLCs, achieving 80% inhibition for *S. aureus*, 40% for *E. coli*, and 30% for MRSA, compared to free ATR (*p* < 0.001). These findings suggest that NLC encapsulation enhances ATR’s antimicrobial efficacy and biofilm suppression. **Conclusions**: This study identified NLCs as successful carriers of ATR, significantly enhancing its antibacterial efficacy and biofilm inhibition capabilities. This formulation, which shows antimicrobial potential against both Gram-positive and Gram-negative bacteria, should be further studied and developed against different resistant microbial strains.

## 1. Introduction

The increasing prevalence of antimicrobial resistance presents a major challenge in modern medicine, driving the need for innovative treatment strategies [1]. The emergence of multidrug-resistant bacterial strains has rendered many conventional antibiotics less effective, leading to increased morbidity, mortality, and healthcare costs [2,3].

One promising approach to addressing this issue is drug repurposing, which explores the potential of existing pharmaceuticals for new therapeutic applications. Statins, which are widely prescribed for cholesterol management, have demonstrated antimicrobial activity, suggesting their potential beyond lipid regulation [4,5].

Atorvastatin (ATR), a commonly used statin, exhibits antibacterial properties against both Gram-positive and Gram-negative bacteria, including drug-resistant strains such as methicillin-resistant *Staphylococcus aureus* (MRSA) and vancomycin-resistant enterococci [6,7]. Studies suggest that ATR exerts its antimicrobial effects by disrupting bacterial membrane integrity, interfering with lipid metabolism, and modulating host immune responses [8]. ATR has also been reported to enhance the antibacterial activity of platelets and exhibit synergistic effects when combined with conventional antibiotics, further supporting its potential as an adjunct antimicrobial therapy [9]. Research has also indicated that ATR can inhibit bacterial biofilm formation, which is a major factor contributing to chronic infections and antibiotic resistance [10,11].

The antimicrobial activity of ATR is believed to be mediated by multiple mechanisms. One proposed pathway is the inhibition of HMG-CoA reductase, which leads to the reduced synthesis of isoprenoid intermediates such as farnesyl pyrophosphate (Fpp) and geranyl pyrophosphate (GGpp). These molecules are essential for post-translational prenylation of proteins involved in bacterial survival, host immune modulation, and pathogen invasion [12,13].

Additionally, ATR-induced depletion of isoprenoids affects the localization and function of small GTPases such as CDC42, which play a key role in bacterial adhesion and host cell invasion. By interfering with these processes, ATR not only disrupts bacterial viability but also modulates host–pathogen interactions, further enhancing its antimicrobial potential [13]. By interfering with these processes, ATR not only disrupts bacterial viability but also modulates host-pathogen interactions, further enhancing its antimicrobial potential.

Although ATR has antibacterial potential, its clinical use is limited by poor solubility, low bioavailability, and rapid metabolism, thus reducing its effectiveness [14]. To overcome these challenges, nanotechnology-based drug delivery systems have gained attention owing to their ability to improve therapeutic efficacy by enhancing drug stability, solubility, and targeted delivery. Nanocarriers can protect drugs from degradation, prolong circulation time, and facilitate controlled release, thereby optimizing their pharmacokinetic and pharmacodynamic profiles [15,16]. These advantages have made nanotechnology a valuable tool for antimicrobial drug development, offering new strategies to combat resistant bacterial infections [17,18,19].

Among the existing nanocarrier systems, nanostructured lipid carriers (NLCs) have been developed as second-generation lipid nanoparticle systems to address the limitations of solid lipid nanoparticles (SLNs), such as their low drug-loading capacity and unstable formulation. NLCs incorporate both solid and liquid lipids, which introduce structural imperfections that enhance drug encapsulation, controlled release, and stability [20,21]. Among the different nanocarrier systems, NLCs were specifically chosen for this study due to their ability to overcome limitations associated with other drug delivery systems, such as polymeric nanoparticles, liposomes, and SLNs. While liposomes and polymeric nanoparticles offer biocompatibility and controlled release, they often suffer from stability issues and high production costs [22,23,24]. SLNs, although structurally similar to NLCs, have a highly ordered crystalline matrix that restricts drug loading and may lead to premature drug expulsion [21]. In contrast, NLCs incorporate both solid and liquid lipids, which introduce structural imperfections that enhance drug encapsulation, controlled release, and stability [22].

These carriers are composed of solid lipids (e.g., triglycerides, fatty acids, waxes, and steroids), liquid lipids (e.g., medium-chain triglycerides, oils, and fatty acids), and surfactants for stabilization [25]. This unique composition prevents the formation of a highly ordered crystalline matrix, reducing drug expulsion and aggregation while improving long-term stability and bioavailability [20]. Additionally, NLCs enhance drug retention, prolong the release duration, and improve absorption, making them suitable for oral, topical, and targeted drug delivery applications [26,27,28]. Various formulation techniques have been employed to optimize the physicochemical properties of NLCs, including high-pressure homogenization, emulsification, solvent evaporation, ultrasonication, and microemulsion-based methods. These methods allow for precise control of particle size, surface charge, drug loading, and stability, which are critical factors that influence the efficiency of drug delivery [25,29].

NLCs have been successfully applied for the delivery of a wide range of pharmaceuticals, including anticancer drugs, anti-inflammatory agents, and antimicrobials [27,30,31,32]. Their ability to enhance drug penetration across biological barriers, improve intracellular uptake, and enable sustained release makes them a promising approach to repurposing ATR as an antimicrobial agent. This study aimed to develop and evaluate ATR-loaded nanostructured lipid carriers (ATR-NLCs) as a novel antimicrobial formulation. ATR-NLCs were prepared using high-pressure homogenization and ultrasonication, and their antimicrobial potential was assessed against gram-positive (MRSA and *S. aureus*) and gram-negative (*E. coli*) bacteria. The incorporation of ATR into NLCs is expected to improve drug solubility, stability, and antibacterial activity, thus offering a more effective approach to combating drug-resistant bacterial infections. By integrating nanomedicine with antimicrobial therapy, this research explores ATR-NLCs as an innovative drug delivery system with potential applications in overcoming antibiotic resistance and addressing a critical challenge in modern healthcare.

## 2. Results

### 2.1. Development and Characterization of NLC Formulations

The method of preparing the NLC was optimized by adjusting the ratio of solid to liquid lipids and the surfactant concentration. The effects of formulation composition on particle size and polydispersity index (PDI) are presented in Table 1. NLC formulation D had a favorable particle size of 130 ± 8.39 nm and PDI of 0.3 ± 0.5, so it was selected for ATR loading.

The physicochemical characteristics of blank NLC and ATR-NLC are summarized in Table 2. The blank NLC had an average particle size of 130 ± 8.39 nm, while the average particle size of the encapsulated NLC was 142 ± 52.20 nm. The average encapsulation efficiency of the NLC formulations, calculated using the indirect method, was 94%.

### 2.2. In Vitro Release Studies

**Figure 1:**
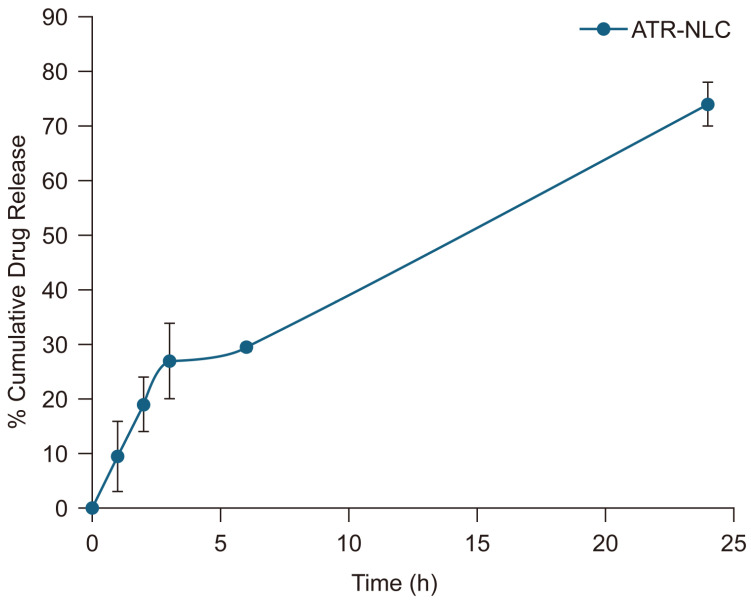
In vitro ATR release study using the dialysis bag method. After 3 h, 27 ± 6% of ATR was released, followed by an almost steady release phase, reaching 74 ± 3% at the 24 h mark. Each value represents the mean ± SD.

The in vitro release data shown in Figure 1 reveals a moderate release profile, with an average of 27 ± 6% ATR released within the first 3 h, followed by an almost steady release phase, reaching 74 ± 3% released at the 24 h mark.

### 2.3. Thermal Analysis

**Figure 2:**
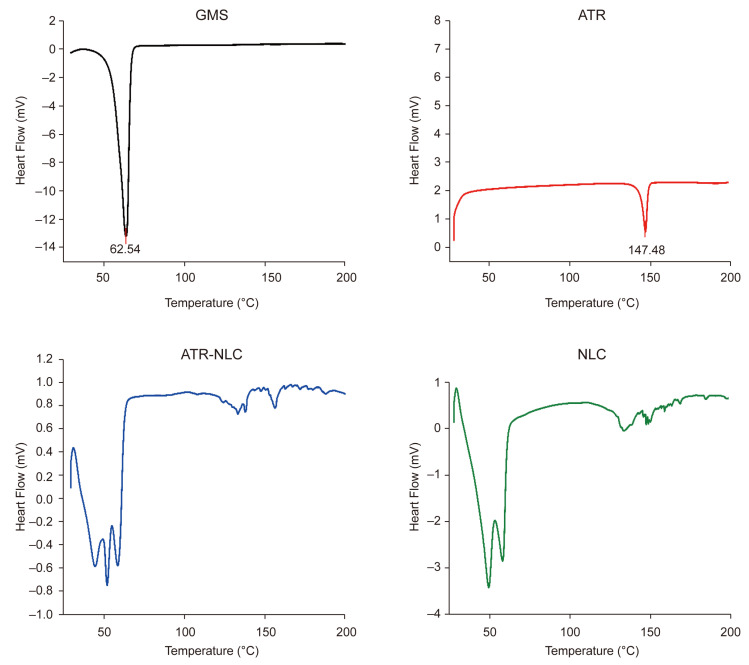
DSC thermograms of GMS, ATR, ATR-NLC, and blank NLC. The ATR thermogram exhibits an endothermic peak at 147.48 °C, corresponding to its melting point and confirming the crystalline nature of the drug. The GMS thermogram shows a sharp endothermic peak at 62.54 °C. In contrast, the thermograms of the lyophilized NLC and ATR-NLC display endothermic peaks around 50 °C, indicating a slight shift in the GMS peak, suggesting interactions between the lipid matrix and the drug.

Figure 2 shows a thermogram of lyophilized GMS, ATR, ATR-NLC, and NLC. The ATR thermogram reveals an endothermic peak at 147.48 °C, corresponding to its melting point and indicating the crystallinity of the drug. On the other hand, the GMS thermogram reveals a sharp endothermic peak at 62.54 °C. The thermograms of the lyophilized NLC and ATR-NLC reveal endothermic peaks around 50 °C, representing a slightly shifted peak of GMS.

**Figure 3:**
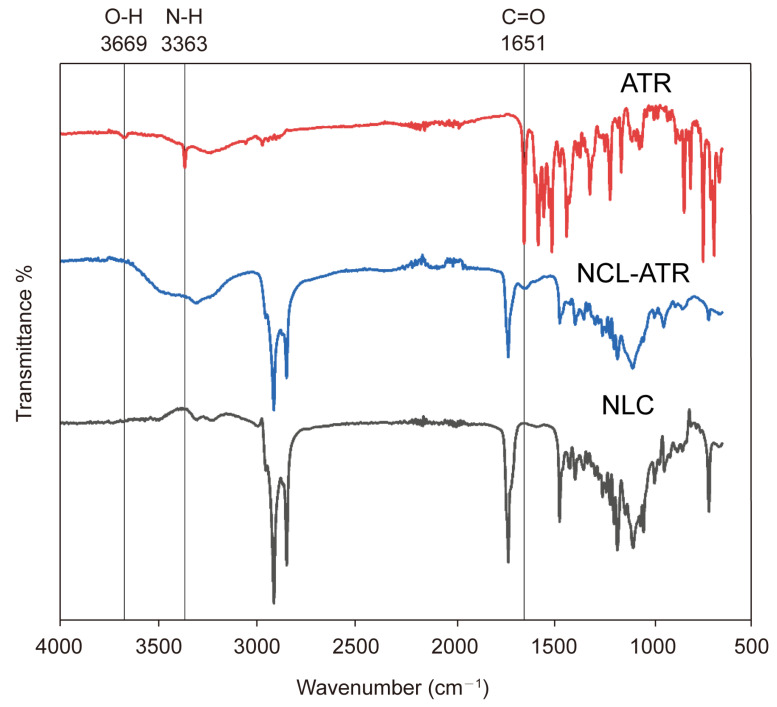
FTIR spectra of ATR, ATR-NLC, and blank NLC. The ATR spectrum exhibits characteristic peaks at 3669 cm^−1^ (O-H), 3363 cm^−1^ (N-H stretching), 3250.4 cm^−1^ (O-H stretching), 2968.87 cm^−1^ (C-H stretching), and 1650 cm^−1^ (C=O stretching of amide). In contrast, the ATR-NLC spectrum shows the disappearance of the ATR peaks at 3669 cm^−1^ and 3363 cm^−1^, along with a reduction in intensity at 1650 cm^−1^, indicating potential molecular interactions between ATR and the lipid matrix.

The Fourier Transform Infrared (FTIR) spectra of the ATR, blank NLC, and encapsulated NLC are presented in Figure 3. The ATR spectrum revealed characteristic peaks at 3669 cm^−1^ (O-H), 3363 cm^−1^ (N-H stretching), 3250.4 cm^−1^ (O-H stretching), 2968.87 cm^−1^ (C-H stretching), and 1650 cm^−1^ (C=O stretching of amides). The spectrum of the ATR-NLC showed the disappearance of the ATR peaks at 3669 cm^−1^ and 3363 cm^−1^, in addition to a reduction in the 1650 cm^−1^ peak compared with the pure ATR spectra, suggesting a molecular interaction between ATR and the lipids.

### 2.4. Morphology: Transmission Electron Microscopy (TEM)

**Figure 4:**
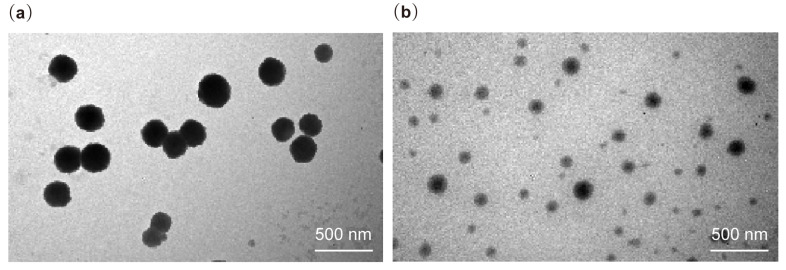
TEM images showing the morphologies of (**a**) ATR-NLC and (**b**) blank NLC. The images reveal well-dispersed, non-aggregating, spherical nanoparticles, highlighting their morphology and nanoscale structure.

The morphology of the NLC and ATR-NLC formulations, shown in Figure 4, reveals non-aggregating spherical nanoparticles, highlighting their morphological characteristics. The size observed is consistent with the particle size measurements obtained using the Zetasizer Nano ZS90 (Malvern Panalytical, Malvern, UK).

### 2.5. Sensitivity Testing Using Agar Well Diffusion Method

The agar well diffusion results (Table 3) demonstrate that the ATR-NLCs exhibit broad-spectrum antimicrobial activity against both Gram-positive and Gram-negative bacteria. The largest zone of inhibition was observed against *S. aureus* (2.3 cm), followed closely by *E. coli* (2.2 cm), with MRSA showing a relatively smaller zone of inhibition (1.8 cm).

### 2.6. Growth Curve Analysis Using Microbroth Dilution Assay

**Figure 5:**
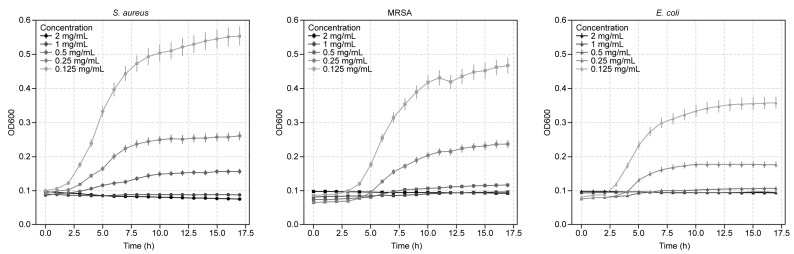
Growth curves of *S. aureus*, MRSA, and *E. coli* in the presence of ATR and NLC formulations. Bacterial cultures (adjusted to a 0.5 McFarland standard) were incubated for 18 h with serially diluted ATR-NLCs (2–0.125 mg/mL) and bacteria only as a control. OD was measured every hour for 18 h to monitor bacterial growth. Higher concentrations of ATR-NLC (2 mg/mL) consistently suppressed bacterial growth more effectively, with *S. aureus* showing the highest sensitivity, followed by *E. coli* and MRSA. Statistical analysis (paired *t*-test) confirmed significant inhibition (*p* < 0.001) at higher ATR-NLC concentrations for all three strains compared to the untreated control, highlighting that the encapsulated ATR is primarily responsible for the observed growth inhibition.

Figure 5 presents the growth curves showing the antimicrobial activity of NLC against the three bacterial strains over 18 h. The graphs display the Optical density (OD) measurements over time for *S. aureus*, MRSA, and *E. coli*. Each curve represents different concentrations of NLCs (2 mg/mL, 1 mg/mL, 0.5 mg/mL, 0.25 mg/mL, and 0.125 mg/mL) and a bacteria control, with error bars indicating the standard deviation. Bacterial strains treated with higher concentrations of ATR-NLCs consistently exhibited greater growth inhibition.

**Figure 6:**
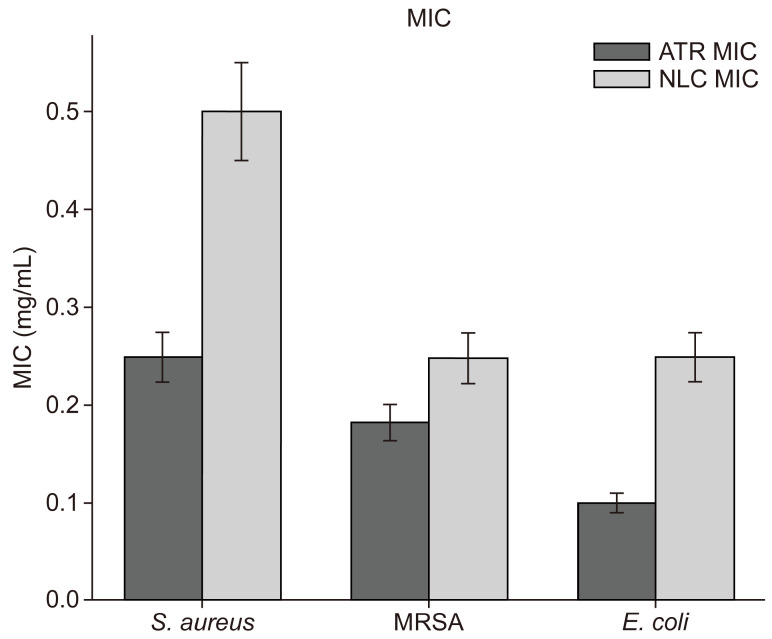
MIC comparison between ATR and ATR-NLCs against *S. aureus*, MRSA, and *E. coli.* Data represents mean ± SD from three independent experiments (*n* = 3). Error bars represent standard deviation (SD).

The minimum inhibitory concentration (MIC) values are shown at the top of each bar in Figure 6. The use of ATR resulted in lower MIC values (0.1–0.25 mg/mL) than ATR-NLC (0.25–0.5 mg/mL). The MIC values for MRSA showed the smallest difference between formulations (1.37-fold increase), whereas the difference was the largest for *E. coli* (2.5-fold increase), suggesting bacterial strain-specific responses to the NLC delivery system.

### 2.7. Biofilm Formation Using Crystal Violet Assay

**Figure 7:**
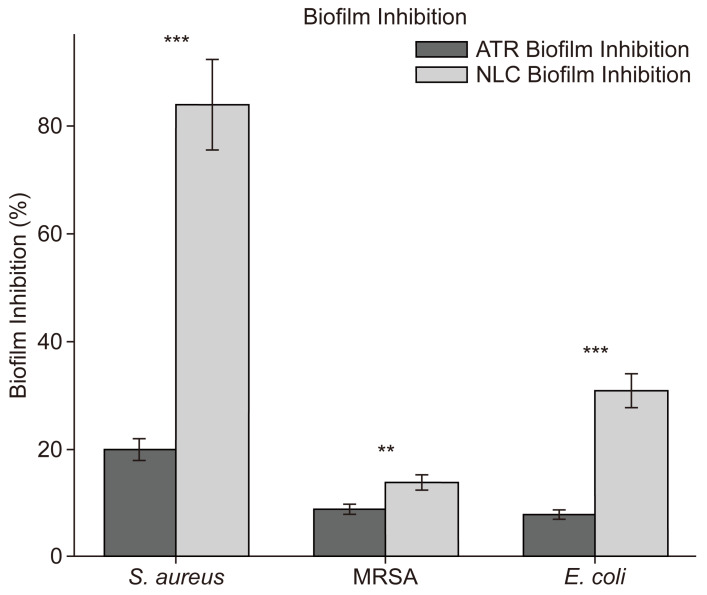
Biofilm inhibition comparison (%) between ATR and ATR-NLC against *S. aureus*, MRSA, and *E. coli.* Data are shown as the mean ± SD from three independent experiments (*n* = 3). Paired *t*-tests were performed to compare ATR-NLC and free ATR groups.Statistical significance is indicated as follows: ** *p* < 0.01, *** *p* < 0.001. Error bars represent standard deviation (SD).

In the biofilm inhibition results presented in Figure 7, for *S. aureus*, ATR and ATR-NLC achieved approximately 20% and 80% biofilm inhibition, respectively, with the most significant difference observed *(p* < 0.001). For MRSA, ATR- and ATR-NLC achieved approximately 10% and 30% biofilm inhibition, respectively. For *E. coli*, ATR- and ATR-NLC achieved approximately 20% and 40% biofilm inhibition, respectively. Significant differences were observed for MRSA and *E. coli* (*p* < 0.01) and (*p* < 0.001, respectively).

## 3. Discussion

The development and characterization of ATR-NLC demonstrate promising potential for enhancing the delivery and efficacy of ATR. The optimized formulation exhibited favorable physicochemical properties, including a particle size of 142 ± 52.20 nm, a PDI of 0.4 ± 0.05, a zeta potential of −31 ± 1.41 mV, and a high encapsulation efficiency of 94%. Previous studies on NLCs showed that particle sizes below 200 nm and zeta potentials above −30 mV are associated with improved stability and cellular uptake [33]. As illustrated in Table 1, the optimization process highlights the importance of the lipid composition and surfactant concentration in achieving the desired NLC characteristics. A higher ratio of GMS to Labrasol resulted in micro-sized particles, which might be attributed to the increased crystallinity and higher viscosity of the lipid matrix hindering the formation of nanosized structures [17]. In contrast, an equal ratio of GMS to Labrasol produced nanosized particles, possibly due to improved lipid dispersion and stabilization within the formulation [34]. The final formulation (Formulation D), which had a 50:50 ratio of GMS to Labrasol and incorporated 2% Tween 80 as a surfactant, yielded the most favorable particle size and PDI. This highlights the critical role of formulation in the successful development and performance of nanoparticle-based drug delivery systems [34,35].

The particle size increased from 130 ± 8.39 nm for blank NLC to 142 ± 52.20 nm for ATR-NLC, likely due to the incorporation of the drug (0.1%) in the lipid matrix. However, the relatively large standard deviation in the size of the ATR-NLC (±52.20 nm) suggests some variability in the formulation process, which may require further optimization. This variability is not uncommon in NLC formulations and has been reported in previous studies [36].

The high EE% of 94% is particularly noteworthy as it suggests that the optimized NLC formulation effectively incorporates ATR within its lipid matrix. This is crucial for enhancing drug solubility and improving bioavailability. GMS, a long-chain monoglyceride, provides a semi-crystalline matrix, whereas Labrasol, a surfactant-based liquid lipid, enhances ATR solubilization within the lipid phase [37].

This high solubility within the lipid matrix ensures that ATR is sufficiently retained in the core, thus minimizing drug loss and enabling efficient encapsulation. Similarly, high EE% values have been reported for ATR-NLC in other studies, such as the study by Elmowafy et al. [6], where an EE% exceeding 87% was achieved.

The in vitro release profile of the ATR-NLC demonstrates a biphasic pattern characterized by an initial burst release followed by a sustained release phase. The initial burst release, in which approximately 27% of ATR was released within the first 3 h, can be attributed to drug molecules adsorbed on or near the surface of the NLCs. This rapid initial release is beneficial for achieving therapeutic concentration quickly. The subsequent sustained release phase, which reaches 70% cumulative release at 24 h, indicates a controlled release pattern that could potentially maintain therapeutic drug levels over an extended period [38]. 

The observed release profile aligns with the findings of several studies, which reported a similar biphasic release pattern for NLCs, with an initial burst release followed by a more gradual and sustained release [6,39,40]. This release behavior is advantageous for maintaining therapeutic drug concentrations while potentially reducing dosing frequency. The moderate release rate observed in this study suggests that the NLC formulation effectively encapsulates ATR and controls its release. This controlled release pattern is likely due to the unique structure of NLCs, which combines solid and liquid lipids to create imperfections in the lipid matrix, allowing for better drug incorporation and controlled release [41]. However, it is important to note that in vitro release studies may not fully predict the in vivo performance. Factors such as physiological conditions, enzymatic degradation, and absorption mechanisms can influence actual drug release and bioavailability in vivo.

DSC results, depicted in Figure 2, reveal clear changes in the thermal behavior of ATR when incorporated into the NLC formulation. The ATR thermogram exhibits an endothermic peak at 147.48 °C, corresponding to its melting point, indicating the crystalline nature of the pure drug. This observation aligns with previous studies reporting similar melting points for crystalline ATR, confirming its stability in the formulation [42]. GMS displayed a sharp endothermic peak at 62.54 °C, which is characteristic of its melting transition. Notably, the lyophilized NLC and ATR-NLC formulations demonstrated shifted GMS endothermic peaks around 50 °C, which can be attributed to interactions between the solid lipid, liquid lipid, and surfactant within the nanostructured matrix, as well as the reduction in the particle size to the nanoscale. A comparable finding was reported by Ghanem et al. [40]. This peak shift suggests a significant alteration in the thermal properties of the GMS, potentially indicating a less ordered or amorphous structure. The absence of ATR’s characteristic melting peak in the ATR-NLC thermogram suggests that the drug was successfully incorporated into the lipid matrix, likely in an amorphous or molecularly dispersed state. This transformation from crystalline to amorphous is crucial as it can enhance drug solubility and bioavailability, which are often limitations for poorly soluble drugs, such as ATR [40,43].

FTIR spectroscopy was used to explore the changes within the NLC formulations and interactions between the drug and lipids. The appearance, shift, or disappearance of a peak in the FTIR spectrum may indicate an interaction at the molecular level. The ATR spectrum in Figure 3 shows characteristic peaks at 3669 cm^−1^ (O-H), 3363 cm^−1^ (N-H stretching), 3250.4 cm^−1^ (O-H stretching), 2968.87 cm^−1^ (C-H stretching), and 1650 cm^−1^ (C=O stretching of amide), which have been reported in several studies [40,44,45]. Compared to the pure ATR spectra, the spectrum of the ATR-NLC shows the disappearance of the ATR peaks at 3669 cm^−1^ and 3363 cm^−1^ and a reduction at 1650 cm^−1^, which suggests a molecular interaction between ATR and the lipids.

TEM analysis, illustrated in Figure 4, provided essential information about the morphology and size distribution of the NLC and ATR-NLC formulations. The images reveal spherical nanoparticles with no visible aggregation, indicating stable formulation properties. This spherical morphology is typical for NLCs and has been reported in numerous studies on lipid-based nanocarriers [23]. The observed particle sizes in the TEM images are consistent with the measurements obtained via DLS (Zetasizer), confirming a uniform nanoparticle size distribution. This consistency enhances the reliability of the particle size characterization and suggests a well-controlled formulation process. The absence of visible drug crystals or aggregates in the TEM images further corroborates the DSC findings, supporting the hypothesis that ATR is homogeneously dispersed within the lipid matrix. This uniform distribution is crucial for maintaining the stability and controlled-release properties of formulations.

In conclusion, both thermal analysis and morphological characterization provide complementary evidence of successful ATR incorporation into the NLC system. The shift in thermal behavior and the formation of uniform spherical nanoparticles suggest a well-designed formulation with the potential for improved drug delivery. These findings align with the existing literature on lipid-based nanocarriers intended to enhance the delivery efficacy for drugs with poor water solubility, such as ATR [6,40]. Further studies are needed to explore the in vivo performance and therapeutic implications of these findings.

The agar well diffusion results provide compelling evidence for the broad-spectrum antimicrobial activity of ATR-NLC against both Gram-positive and Gram-negative bacteria. This finding is particularly noteworthy, given the ongoing challenge of developing effective treatments for diseases caused by diverse bacterial pathogens. The observed zones of inhibition demonstrate a clear hierarchy of susceptibility among the tested strains (Table 3). These results are especially significant compared to those of previous studies on ATR’s antimicrobial activity of ATR. For instance, Masadeh et al. [7] reported much higher MICs of ATR against similar strains. The enhanced activity observed in our study suggests that the NLC formulation substantially improves ATR’s antibacterial efficacy due to increased bioavailability and cellular penetration. The effectiveness against MRSA is particularly promising, although it demonstrated a smaller zone of inhibition. MRSA is notoriously difficult to treat owing to its resistance to multiple antibiotics. The fact that ATR-NLC show activity against this strain opens new possibilities for combating antibiotic-resistant infections. Moreover, its comparable efficacy against both Gram-positive (*S. aureus*) and Gram-negative (*E. coli*) bacteria is noteworthy. Many antimicrobial agents struggle to penetrate the outer membrane of Gram-negative bacteria, thereby limiting their effectiveness. The effectiveness of ATR-NLC in inhibiting the growth of *E. coli* and *S. aureus* indicates a potential benefit compared to certain existing treatments. These findings align with recent research on NLCs, which has shown promise in enhancing the antimicrobial activity of various compounds [17,18].

In addition, growth curve analysis revealed concentration-dependent inhibition of bacterial growth in all tested strains. This dose-dependent effect is a hallmark of effective antimicrobial agents and suggests that ATR in ATR-NLC maintains its antibacterial activity, even when encapsulated. Interestingly, while the application of the ATR-NLC resulted in higher MIC values (0.25–0.5 mg/mL) compared to free ATR (0.1–0.25 mg/mL), the NLC formulation still maintained significant antimicrobial activity against all tested strains. This slight increase in MICs when applying the ATR-NLC is not uncommon for nanoformulations and can be attributed to the controlled release properties of NLCs [6,39].

The observed strain-specific responses to the NLC delivery system are particularly noteworthy. MRSA showed the smallest difference in MIC between free ATR and the ATR-NLC (1.37-fold increase), whereas *E. coli* demonstrated the largest difference (2.5-fold increase). This variability suggests that the NLC formulation may interact differently with the cell walls of Gram-positive and Gram-negative bacteria, potentially owing to differences in their structural composition. These findings are consistent with those of previous studies that showed varying degrees of statin efficacy against different bacterial species [46].

In this study, the crystal violet assay revealed that ATR-NLC achieved markedly higher biofilm inhibition than free ATR across all the tested strains. Such increases in inhibition suggest that incorporating ATR into a nanostructured lipid carrier significantly augments its antibiofilm properties, likely through improved drug solubility, extended-release, and enhanced penetration into the biofilm matrix. These findings are consistent with previous reports that statin-loaded nanocarriers can disrupt biofilm integrity more effectively than free statins [42], which is clinically important given the contribution of biofilms to persistent bacterial infections and antibiotic resistance.

In our study, ATR-NLC substantially reduced biofilm formation by both *S. aureus* and *E. coli*. This finding is particularly encouraging for chronic infections. For instance, *S. aureus* commonly causes hospital-acquired infections and can form resistant biofilms on implants and catheters, protecting the bacteria from antibiotics and the immune system [47]. An 80% inhibition of *S. aureus* biofilm by ATR-NLC suggests this formulation could drastically lower the risk of such biofilm establishment. This raises the possibility of using ATR-NLCs as a preventive coating on medical devices or as a local gel in wound care to impede biofilm development in future studies. These improvements are consistent with previous studies showing that nanocarrier-based delivery systems enhance the penetration and efficacy of antimicrobial agents against biofilms [48,49]. Biofilms are highly resistant to antibiotics because of their extracellular polymeric substance (EPS) matrix, which limits drug penetration, facilitates horizontal gene transfer and protects embedded bacteria from environmental stressors [50]. The ability of ATR-NLCs to disrupt biofilms more effectively than free ATR can be attributed to several factors.

1. Improved solubility and stability:The encapsulation of ATR in NLCs enhances its solubility and stability, allowing for higher effective concentrations at the site of infection [51].
2. Controlled drug release:The sustained release profile of ATR-NLCs ensures prolonged exposure of the biofilm to therapeutic concentrations of ATR, which is critical for disrupting mature biofilms [17,18].
3. Enhanced penetration:NLCs are known to penetrate biofilm matrices more effectively than free drugs because of their nanoscale size and lipid composition, which facilitate interactions with bacterial membranes [8,9].

Similarly, simvastatin has shown antibiofilm activity in previous studies. For example, simvastatin–hydroxyapatite coatings were found to inhibit *S. aureus* biofilm formation by disrupting bacterial adhesion and reducing EPS production [28]. However, the performance of ATR-NLCs against both Gram-positive (*S. aureus*, MRSA) and Gram-negative (*E. coli*) bacteria highlights their broader spectrum of activity compared to simvastatin.

Biofilm-associated infections are a major challenge in healthcare because of their resistance to antibiotics, often requiring drug concentrations up to 1000 times higher than those required for planktonic cells [52]. The improvement in biofilm inhibition by ATR-NLCs suggests that this formulation could overcome some of these challenges by enhancing drug delivery and efficacy. The superior efficacy against *S. aureus* is particularly noteworthy given its role in nosocomial infections and its ability to form robust biofilms on medical devices [7]. Similarly, activity against MRSA is critical, as this strain is a leading cause of antibiotic-resistant infections worldwide. One potential clinical use of ATR-NLCs is in wound care and infection prevention [47]. Nanoparticle-based systems have inherent advantages in targeting biofilm-associated infections, as they can interfere with bacterial adhesion, disrupt cell signaling involved in biofilm formation, penetrate the biofilm matrix, and enhance the localized delivery of antimicrobial agents—ultimately weakening the biofilm structure and reducing bacterial survival [53].

Other therapeutic strategies for combating biofilms include combination therapies and quorum-sensing inhibitors [54]. Although these approaches have shown promise, they often require complex formulations or high doses that may lead to toxicity. In contrast, ATR-NLCs offer a simpler yet highly effective strategy by leveraging nanotechnology to enhance the intrinsic antibiofilm properties of ATR.

The results of the crystal violet assay strongly support the potential use of ATR-NLC as an innovative solution for managing biofilm-associated infections. By significantly enhancing ATR’s antibiofilm activity of ATR against both Gram-positive and Gram-negative bacteria, this formulation addresses a critical gap in current antimicrobial therapies. Future studies should focus on elucidating the exact mechanisms underlying this enhanced efficacy and evaluating the in vivo performance of ATR-NLCs in clinically relevant models.

## 4. Materials and Methods

### 4.1. Materials

#### 4.1.1. Chemicals and Reagents

ATR was provided by SPIMACO ADDWAEIH (Riyadh, Saudi Arabia). Labrasol was provided by Gattefosse (Saint-Priest, France). Tween 80 was purchased from Thermo Scientific Chemicals (Waltham, MA, USA), and glyceryl monostearate (GMS) was obtained from BDH Chemicals, Ltd. (Poole, England), and a dialysis bag (MWCO 12–14 kDa) were obtained from SERVAPOR (Heidelberg, Germany). Luria–Bertani (LB) broth and agar were purchased from Oxoid (Hampshire, UK). All other chemicals and solvents were of high analytical grade.

#### 4.1.2. Bacterial Strains

*S. aureus* ATCC 29213, MRSA ATCC 43300, and *E. coli* ATCC 25922 were used in this study.

### 4.2. Preparation of NLC

The NLC formulations were prepared using high-shear homogenization (ULTRA-TURRAX Basic T25 IKA, Wilmington, NC, USA) followed by ultrasonication (Sonics, Newton, CT, USA). Lipid and aqueous phases were prepared separately. The lipid phase consisted of GMS as the solid lipid, which was selected for its amphiphilic nature to enhance ATR encapsulation and maintain the matrix stability. Labrasol, a liquid lipid, was chosen for its high solubilizing capacity for ATR, which allows it to aid in drug incorporation and permeability [55]. To obtain drug-loaded NLC, 0.1% ATR was added to the lipid phase.

The aqueous phase contained Tween 80 as a hydrophilic emulsifier, which was dissolved in distilled water. Tween 80 was used for its hydrophilic–lipophilic balance (HLB), enabling it to contribute to nanoparticle stabilization and prevent aggregation (Table 1).

First, the lipid phase melted at a temperature of 50–55 °C and raised to 10–15 °C above the melting point of the GMS. The aqueous phase was then heated at the same temperature. Once both phases reached equilibrium, the aqueous phase was added dropwise to the lipid phase and thoroughly mixed using a heating magnetic stirrer plate for 10 min. Subsequently, the mixture was homogenized for 30 s at 3000 rpm. It was then ultrasonicated for 5 min, and the device was operated in pulse mode with 20 s on and 10 s off. The resulting emulsions were then cooled to room temperature. For further investigation, the formulations were lyophilized or freshly stored in tightly sealed containers at 4 Â °C. The NLC formulations were lyophilized using a Free Zone console freeze dryer (Labconco, Kansas City, MO, USA) set at −86 °C and a pressure of 0.23 mbar, with mannitol as a cryoprotectant.

### 4.3. Characterization of NLCs

The mean particle size, PDI, and zeta potential of blank NLC and ATR-NLC were measured using a Zetasizer Nano ZS90 (Malvern Panalytical, Malvern, UK). Disposable polystyrene cuvettes were used for particle size measurements, while a folded capillary cell was used to obtain the zeta measurements. The samples were diluted 20 times (1:20, *v*/*v*) with distilled water and measured in triplicate at room temperature.

### 4.4. Encapsulation Efficiency

The ATR amount was analyzed using a UV spectrophotometer at 246 nm (Shimadzu, UV-1800 PC, Kyoto, Japan). A calibration curve was generated using six concentrations ranging from 0 to 25 µg/mL. The resulting correlation coefficient was >0.999.

The encapsulation efficiency (EE%) of ATR-NLC was determined using an indirect method. Vials containing 2 mL of the ATR-NLC preparation were centrifuged for 2 h at 16,000 rpm and 4 °C using a Sigma 3–30 K refrigerated high-speed centrifuge (Osterode am Harz, Germany). The supernatant was diluted to a suitable concentration, and the concentration of free (non-encapsulated) drug was measured spectrophotometrically against a blank. The encapsulation efficiency of ATR was calculated using the following equation:

(1) $$EE\%=\frac{\left(Dt-Df\right)}{Dt}\times 100$$

where *EE*% is the encapsulation efficiency percentage, *Dt* is the total amount of drug added, and *Df* is the amount of free drug in the supernatant after centrifugation.

### 4.5. In Vitro Release Studies

In vitro release studies were performed to assess the drug release patterns using the dialysis bag method. A dialysis bag with a molecular weight cutoff of 12–14 kDa was washed and soaked overnight in phosphate-buffered saline (PBS) at a pH of 6.8. Then, 2 mL of the optimized ATR-NLC formulation was added to the dialysis bag, which was securely closed. The bag was immersed in a 100 mL beaker containing preheated buffer at 37 °C and securely covered. The contents of the beaker were magnetically stirred at 200 rpm. Samples (100 µL) were withdrawn at predetermined time intervals (1, 2, 3, 6, and 24 h). An equal volume of fresh preheated PBS was added to the beaker after each sampling to maintain sink conditions. The concentration of drug released in the samples was determined using a spectrophotometer at 246 nm. All experiments were performed in triplicate, and the results are expressed as the mean ± standard deviation.

### 4.6. Thermal Analysis

Thermal analysis was performed using a differential scanning calorimeter (DSC 4000, PerkinElmer, Shelton, CT, USA). Free ATR, GMS, and lyophilized ATR-NLCs were analyzed; 1 mg samples were placed in aluminum pans and sealed with a lid, along with the standard reference, and thermograms were recorded between 25 °C and 400 °C at a scan rate of 10 °C/min.

### 4.7. Fourier Transform Infrared (FTIR) Spectroscopy

To identify the interactions between the various components used in the preparation of the NLC formulations, an FTIR spectrometer (PerkinElmer, USA) was used in the frequency range of 600–4000 cm^−1^. The spectra of ATR, blank NLC, and encapsulated NLC were recorded.

### 4.8. Morphology Evaluation

The optimized ATR-NLC and blank NLC formulations were visualized using transmission electron microscopy (TEM) to determine NLC morphology. The samples were diluted 50 times with Milli-Q water, after which a drop of the diluted NLC solution was placed on a carbon type-B copper grid (Ted Pella Inc., Redding, CA, USA). The NLCs were allowed to settle for 30 s, and the excess was removed using filtered water strips. Negative staining of settled NLC was performed with 1% phosphotungstic acid in PBS (pH = 7). The grids were then left to dry at room temperature and loaded into a JEM-1010 TEM (JEOL, Tokyo, Japan) operated at an accelerating voltage of 80 kV. Images were recorded using a high-speed read-out side-mounted MegaViewG2 camera (Olympus, Hamburg, Germany) and processed using iTEM version 5.1 software (Olympus Soft Imaging Solutions GmbH, Munster, Germany).

### 4.9. Antibacterial Activity

#### 4.9.1. Sensitivity Testing Using Well Diffusion Method

Overnight cultures of *S. aureus*, MRSA, and *E. coli* in LB broth were adjusted to a 0.5 McFarland standard. ATR-NLC (1 mg/mL) were dissolved in an appropriate solvent (i.e., dimethyl sulfoxide [DMSO]) to obtain the desired test concentration. The suspension was thoroughly mixed by vortexing to ensure solubilization.

Wells of uniform diameter were created in agar plates using a sterile cork borer. The ATR-NLC dispersion (100 μL) was carefully dispensed into the wells. Separate wells were also prepared for the negative control (solvent only) and positive control (standard antibiotics, i.e., oxacillin for *S. aureus*/MRSA and ampicillin for *E. coli*). The agar plates were incubated overnight at an optimal temperature for bacterial growth (37 °C)., oxacillin for MRSA, and ampicillin for *E. coli*). The agar plates were incubated overnight at the optimal temperature for bacterial growth (37 °C). After incubation, the plates were visually examined for inhibition zones around the wells. The diameters of the clear zones (inhibition zones) that formed around each well were measured using a ruler.

#### 4.9.2. Growth Curve Analysis Using Microbroth Dilution Assay

Overnight cultures of *S. aureus*, MRSA, and *E. coli* in LB broth were adjusted to a 0.5 McFarland standard. A serial dilution of 2 mg/mL ATR dissolved in DMSO was prepared to obtain the desired test concentration. The solution was thoroughly mixed by vortexing to ensure solubilization.

Next, 20 μL of each bacterial culture was added to 100 μL of the ATR solution in a 100-well honeycomb plate, along with the blank formulation as a negative control and free ATR as a positive control. The plates were placed in a microplate reader (Bioscreen, Torrance, CA, USA), which allowed continuous monitoring of optical density (OD). The samples were incubated under optimal growth conditions at 37 °C and 595 nm wavelength. The OD of each sample was measured every hour during an 18-h period using a microplate reader.

#### 4.9.3. Crystal Violet Assay

Overnight cultures of *S. aureus*, MRSA, and *E. coli* in LB broth were adjusted to a 0.5 McFarland standard. Serial dilutions of 2 mg/mL ATR dissolved in DMSO were prepared to obtain the desired test concentration. The solution was thoroughly mixed by vortexing to ensure solubilization.

Next, 30 μL of each bacterial culture was added to 170 μL ATR solution (ATR-loaded NLCs or free ATR) and placed in 96-well plates alongside the blank formulation as the negative control. Free ATR was prepared by dissolving ATR in dimethyl sulfoxide (DMSO, 10 mg/mL stock), followed by dilution in Luria–Bertani (LB) broth to a final concentration of 2 mg/mL, ensuring that DMSO did not exceed 1% *v*/*v* in the test wells. Serial dilutions were performed to assess ATR’s effect of ATR on biofilm formation. The plates were incubated at 37 °C for 24 h to allow biofilm formation. After incubation, planktonic cells were removed, and the plates were washed twice with 0.9% NaCl. The plates were dried at room temperature for 15 min. Subsequently, 200 μL of 0.1% crystal violet was added to each well to stain the biofilm. Following staining, the attached crystal violet was dissolved in 200 μL of ethanol–acetone (80:20 *v*/*v*). Finally, a microplate reader (BioTek, Seattle, WA, USA) was used to measure the crystal violet absorbance at 595 nm. All experiments were performed independently in triplicate. The following formula was used for biofilm inhibition calculations:

(2) $$Biofilm\;inhibition\;\%=\frac{Control\;OD}{Control\;OD-Treated\;OD}\times 100$$

### 4.10. Statistical Analysis

The results are presented as mean ± standard deviation (SD) of at least three replicates. The biofilm inhibition data for the ATR and NLC treatments were analyzed using paired *t*-tests to compare the mean inhibition percentages for each bacterium. Statistical significance was determined at a threshold of *p* < 0.05.

## 5. Conclusions

In conclusion, this study demonstrated the potential of ATR-NLCs as a novel antimicrobial formulation that is effective against both Gram-positive and Gram-negative bacteria, including resistant strains. Key findings include the development of ATR-NLCs with optimal physicochemical properties, a controlled release profile indicating sustained antimicrobial activity, and significant antibacterial activity against *Staphylococcus aureus*, MRSA, and *E. coli*. Enhanced biofilm inhibition, particularly against *S. aureus*, and maintained antimicrobial efficacy despite slightly higher MIC values suggest advantages in drug delivery and bioavailability.

Future research should focus on elucidating the mechanisms of enhanced antibiofilm activity, conducting in vivo studies, exploring potential synergistic effects with conventional antibiotics, and optimizing the formulation to improve antimicrobial properties and reduce MIC values. Overall, ATR-NLCs show promise as an innovative approach to combating bacterial infections, including those caused by resistant strains, contributing significantly to nanomedicine and the fight against antimicrobial resistance.

## Figures and Tables

**Table 1:** Characteristics of different NLC formulations.

Formulation	GMS: Labrasol	Surfactant (Tween 80)	Particle Size	PDI
A	70:30	1%	>1 µm	---
B	70:30	2%	>1 µm	---
C	50:50	1%	>300 nm	0.4
D ^1^	50:50	2%	130 ± 8.39 nm	0.3 ± 0.5

^1^ optimum formulation used in the study.

**Table 2:** Characteristics of blank NLC and ATR-NLC.

Formulation	Particle Size (nm)	PDI	Zeta Potential (mV)	EE%
Blank NLC	130 ± 8.39	0.3 ± 0.5	−35 ± 0.5	---
ATR-NLC	142 ± 52.20	0.4 ± 0.05	−31 ± 1.41	94%

**Table 3:** Zone of inhibition measurements, mean ± SD (in cm), for ATR-NLC against Gram-positive (*S. aureus* and MRSA) and gram-negative (*E. coli*) bacteria using the agar well diffusion method.

Bacteria	Zone of Inhibition (cm)
*S. aureus*	2.3 ± 0.1
MRSA	1.8 ± 0.1
*E. coli*	2.2 ± 0.1

## Data Availability

The authors confirm that the data supporting the findings of this study are available within the article.

## References

[1] World Health Organization (2022). Global Antimicrobial Resistance and Use Surveillance System (GLASS) Report 2022.

[2] Su J., Wang Y., Zhong W., Wang M., Wang Y. (2024). A retrospective study on the effect of statins on mortality and antimicrobial resistance among patients with *Staphylococcus aureus* bloodstream infection. Arch. Med. Sci. AMS.

[3] O’Neill J. (2016). Tackling Drug-Resistant Infections Globally: Final Report and Recommendations.

[4] Ashburn T.T., Thor K.B. (2004). Drug repositioning: Identifying and developing new uses for existing drugs. Nat. Rev. Drug Discov..

[5] Hennessy E., Adams C., Reen F.J., O’Gara F. (2016). Is there potential for repurposing statins as novel antimicrobials?. Antimicrob. Agents Chemother..

[6] Elmowafy M., Ibrahim H.M., Ahmed M.A., Shalaby K., Salama A., Hefesha H. (2017). Atorvastatin-loaded nanostructured lipid carriers (NLCs): Strategy to overcome oral delivery drawbacks. Drug Deliv..

[7] Masadeh M., Mhaidat N., Alzoubi K., Al-Azzam S., Alnasser Z. (2012). Antibacterial activity of statins: A comparative study of atorvastatin, simvastatin, and rosuvastatin. Ann. Clin. Microbiol. Antimicrob..

[8] Davuluri K.S., Singh A.K., Singh A.V., Chaudhary P., Raman S.K., Kushwaha S., Singh S.V., Chauhan D.S. (2023). Atorvastatin potentially reduces mycobacterial severity through its action on Lipoarabinomannan and Drug Permeability in Granulomas. Microbiol. Spectr..

[9] Hannachi N., Fournier P.-E., Martel H., Habib G., Camoin-Jau L. (2021). Statins potentiate the antibacterial effect of platelets on *Staphylococcus aureus*. Platelets.

[10] Manalo R.V.M., Josol V.J.D., Gloriani N.G. (2017). The differential effects of atorvastatin co-administered with ampicillin on the bacterial growth and biofilm formation of *Staphylococcus aureus*. Curr. Med. Res. Pract..

[11] Sajal Sarabhai S.S., Dhaliwal L., Neena Capalash N.C., Prince Sharma P.S. (2015). Effect of atorvastatin and rosuvastatin on quorum sensing, biofilm formation and bacterial motilities of Pseudomonas aeruginosa. Int. J. Pharma Bio Sci..

[12] Zhang F.L., Casey P.J. (1996). Protein prenylation: Molecular mechanisms and functional consequences. Annu. Rev. Biochem..

[13] Evans M.D., McDowell S.A. (2021). Pleiotropic effects of statins: New therapeutic approaches to chronic, recurrent infection by *Staphylococcus aureus*. Pharmaceutics.

[14] Sirtori C.R. (2014). The pharmacology of statins. Pharmacol. Res..

[15] Cheng X., Xie Q., Sun Y. (2023). Advances in nanomaterial-based targeted drug delivery systems. Front. Bioeng. Biotechnol..

[16] Majumder J., Taratula O., Minko T. (2019). Nanocarrier-based systems for targeted and site specific therapeutic delivery. Adv. Drug Deliv. Rev..

[17] Zhang J., Liu M., Guo H., Gao S., Hu Y., Zeng G., Yang D. (2024). Nanotechnology-driven strategies to enhance the treatment of drug-resistant bacterial infections. Wiley Interdiscip. Rev. Nanomed. Nanobiotechnol..

[18] Singh R., Smitha M., Singh S.P. (2014). The role of nanotechnology in combating multi-drug resistant bacteria. J. Nanosci. Nanotechnol..

[19] Makabenta J.M.V., Nabawy A., Li C.-H., Schmidt-Malan S., Patel R., Rotello V.M. (2021). Nanomaterial-based therapeutics for antibiotic-resistant bacterial infections. Nat. Rev. Microbiol..

[20] Müller R.H., Shegokar R., Keck C.M. (2011). 20 years of lipid nanoparticles (SLN & NLC): Present state of development & industrial applications. Curr. Drug Discov. Technol..

[21] Müller R., Radtke M., Wissing S. (2002). Nanostructured lipid matrices for improved microencapsulation of drugs. Int. J. Pharm..

[22] Das S., Chaudhury A. (2011). Recent advances in lipid nanoparticle formulations with solid matrix for oral drug delivery. AAPS PharmSciTech.

[23] Khan S., Sharma A., Jain V. (2023). An overview of nanostructured lipid carriers and its application in drug delivery through different routes. Adv. Pharm. Bull..

[24] Salvi V., Pawar P. (2019). Nanostructured lipid carriers (NLC) system: A novel drug targeting carrier. J. Drug Deliv. Sci. Technol..

[25] Gomaa E., Fathi H.A., Eissa N.G., Elsabahy M. (2022). Methods for preparation of nanostructured lipid carriers. Methods.

[26] Beloqui A., Solinís M.Á., Rodríguez-Gascón A., Almeida A.J., Préat V. (2016). Nanostructured lipid carriers: Promising drug delivery systems for future clinics. Nanomed. Nanotechnol. Biol. Med..

[27] Haider M., Abdin S.M., Kamal L., Orive G. (2020). Nanostructured lipid carriers for delivery of chemotherapeutics: A review. Pharmaceutics.

[28] Sabale V., Jiwankar M. (2023). Nanostructured Lipid Carriers in Chemotherapeutics: An Overview. Indian J. Pharm. Educ. Res..

[29] Viegas C., Patrício A., Prata J., Nadhman A., Chintamaneni P., Fonte P. (2023). Solid Lipid Nanoparticles vs. Nanostructured Lipid Carriers: A Comparative Review. Pharmaceutics.

[30] Sütő B., Berkó S., Kozma G., Kukovecz Á., Budai-Szűcs M., Erős G., Kemény L., Sztojkov-Ivanov A., Gáspár R., Csányi E. (2016). Development of ibuprofen-loaded nanostructured lipid carrier-based gels: Characterization and investigation of in vitro and in vivo penetration through the skin. Int. J. Nanomed..

[31] Cortesi R., Valacchi G., Muresan X.M., Drechsler M., Contado C., Esposito E., Grandini A., Guerrini A., Forlani G., Sacchetti G. (2017). Nanostructured lipid carriers (NLC) for the delivery of natural molecules with antimicrobial activity: Production, characterisation and in vitro studies. J. Microencapsul..

[32] Bondì M.L., Craparo E.F., Giammona G., Cervello M., Azzolina A., Diana P., Martorana A., Cirrincione G. (2007). Nanostructured lipid carriers-containing anticancer compounds: Preparation, characterization, and cytotoxicity studies. Drug Deliv..

[33] Albekery M., Alharbi K., Alarifi S., Ahmad D., Omer M., Massadeh S., Yassin A. (2017). Optimization of a nanostructured lipid carriers system for enhancing the biopharmaceutical properties of valsartan. Dig. J. Nanomater. Biostruct..

[34] Apostolou M., Assi S., Fatokun A.A., Khan I. (2021). The effects of solid and liquid lipids on the physicochemical properties of nanostructured lipid carriers. J. Pharm. Sci..

[35] Ghasemiyeh P., Mohammadi-Samani S. (2018). Solid lipid nanoparticles and nanostructured lipid carriers as novel drug delivery systems: Applications, advantages and disadvantages. Res. Pharm. Sci..

[36] Kim M.-H., Kim K.-T., Sohn S.-Y., Lee J.-Y., Lee C.H., Yang H., Lee B.K., Lee K.W., Kim D.-D. (2019). Formulation and evaluation of nanostructured lipid carriers (NLCs) of 20 (S)-protopanaxadiol (PPD) by Box-Behnken design. Int. J. Nanomed..

[37] Pandit A.P., Chavan T.T., Khandelwal K.R. (2015). Enhancement of solubility, dissolution rate and bioavailability of atorvastatin using solid lipid: In vitro and in vivo characterization. J. Pharm. Investig..

[38] Mahor A.K., Singh P.P., Gupta R., Bhardwaj P., Rathore P., Kishore A., Goyal R., Sharma N., Verma J., Rosenholm J.M. (2023). Nanostructured lipid carriers for improved delivery of therapeutics via the oral route. J. Nanotechnol..

[39] Unnisa A., Chettupalli A.K., Alazragi R.S., Alelwani W., Bannunah A.M., Barnawi J., Amarachinta P.R., Jandrajupalli S.B., Elamine B.A., Mohamed O.A. (2023). Nanostructured lipid carriers to enhance the bioavailability and solubility of ranolazine: Statistical optimization and pharmacological evaluations. Pharmaceuticals.

[40] Ghanem H.A., Nasr A.M., Hassan T.H., Elkhoudary M.M., Alshaman R., Alattar A., Gad S. (2021). Comprehensive study of atorvastatin nanostructured lipid carriers through multivariate conceptualization and optimization. Pharmaceutics.

[41] Sreedhar R., Kumar V., Bhaskaran Pillai A., Mangalathillam S. (2019). Omega-3 Fatty Acid Based Nanolipid Formulation of Atorvastatin for Treating Hyperlipidemia. Adv. Pharm. Bull..

[42] Raschi E., Casula M., Cicero A.F., Corsini A., Borghi C., Catapano A. (2023). Beyond statins: New pharmacological targets to decrease LDL-cholesterol and cardiovascular events. Pharmacol. Ther..

[43] KM A.S., Angolkar M., Rahamathulla M., Thajudeen K.Y., Ahmed M.M., Farhana S.A., Shivanandappa T.B., Paramshetti S., Osmani R.A.M., Natarajan J. (2024). Box-Behnken Design-Based Optimization and Evaluation of Lipid-Based Nano Drug Delivery System for Brain Targeting of Bromocriptine. Pharmaceuticals.

[44] Ibesh S., Bitar Y., Trefi S. (2023). A New method for simultaneous qualitative and quantitative determination of amlodipine besylate and atorvastatin calcium in bulk and pharmaceutical formulations using transmission FT-IR spectroscopy. Heliyon.

[45] Sharma M., Isha M. (2019). Surface stabilized atorvastatin nanocrystals with improved bioavailability, safety and antihyperlipidemic potential. Sci. Rep..

[46] Kamińska M., Aliko A., Hellvard A., Bielecka E., Binder V., Marczyk A., Potempa J., Delaleu N., Kantyka T., Mydel P. (2019). Effects of statins on multispecies oral biofilm identify simvastatin as a drug candidate targeting Porphyromonas gingivalis. J. Periodontol..

[47] Tuon F.F., Suss P.H., Telles J.P., Dantas L.R., Borges N.H., Ribeiro V.S.T. (2023). Antimicrobial treatment of *Staphylococcus aureus* biofilms. Antibiotics.

[48] Ahsan A., Thomas N., Barnes T.J., Subramaniam S., Loh T.C., Joyce P., Prestidge C.A. (2024). Lipid Nanocarriers-Enabled Delivery of Antibiotics and Antimicrobial Adjuvants to Overcome Bacterial Biofilms. Pharmaceutics.

[49] Wang D.-Y., Van der Mei H.C., Ren Y., Busscher H.J., Shi L. (2020). Lipid-based antimicrobial delivery-systems for the treatment of bacterial infections. Front. Chem..

[50] Hu X., Kang F., Yang B., Zhang W., Qin C., Gao Y. (2019). Extracellular polymeric substances acting as a permeable barrier hinder the lateral transfer of antibiotic resistance genes. Front. Microbiol..

[51] Elmowafy M., Al-Sanea M.M. (2021). Nanostructured lipid carriers (NLCs) as drug delivery platform: Advances in formulation and delivery strategies. Saudi Pharm. J..

[52] Penesyan A., Nagy S.S., Kjelleberg S., Gillings M.R., Paulsen I.T. (2019). Rapid microevolution of biofilm cells in response to antibiotics. NPJ Biofilms Microbiomes.

[53] Jing G., Hu C., Fang K., Li Y., Wang L. (2024). How nanoparticles help in combating chronic wound biofilm infection?. Int. J. Nanomed..

[54] Hawas S., Verderosa A.D., Totsika M. (2022). Combination therapies for biofilm inhibition and eradication: A comparative review of laboratory and preclinical studies. Front. Cell. Infect. Microbiol..

[55] Nasr M., Ramzy M., Abdel-moneum R., Abdel-Rashid R.S. (2025). Optimization of nano-structured lipid carriers for enhanced salbutamol delivery via buccal mucoadhesive film. J. Drug Deliv. Sci. Technol..

